# Defect Detection Sensitivity of Bubble-Point-Type Tests for Sterilizing-Grade Membrane Cartridge Filters

**DOI:** 10.3390/membranes13010088

**Published:** 2023-01-10

**Authors:** Sal Giglia, Anand Alembath, Joseph Hersey

**Affiliations:** EMD Millipore Corporation, an Affiliate of Merck, Bedford, MA 01730, USA

**Keywords:** sterilizing filter, integrity test, bacterial retention, bubble point, filter defect

## Abstract

Bubble point tests are widely used for assessing the integrity of sterilizing-grade membrane cartridge filters. While many authors have considered the limitations of bubble point tests as applied to cartridge filters, the level of bacterial retention assurance provided by this test as conducted with automated integrity testers (AITs) has not, until now, been quantified. Contrary to the notion that filter leaks result in a depressed bubble point, it was shown that the bubble point as reported by AITs was insensitive to defect size up until the point where the AIT either determined a gross leak failure or was not able to return a valid result. For the three AITs used in this study, the minimum laser hole defect diameter in 10-inch (25.4 cm) sterilizing-grade cartridge filters that resulted in a failing bubble point test was between about 30 and 60 µm, depending on the filter type and test conditions. These defect sizes were associated with bacterial log reduction values in the 4.0 to 4.5 range. This study supports the generally recommended practice of pairing the bubble point test (which does confirm proper pore size rating) with a complementary gas–liquid diffusion test (better suited for detecting defects) to achieve a more comprehensive assessment of filter integrity.

## 1. Introduction

Assurance of the bacterial retention performance of sterilizing-grade filters relies on a multitiered approach that includes the filter manufacturing process, product validation, membrane release tests, device release tests, end-user validation, and tests including integrity tests. Integrity tests of filters designed for sterile filtration are routinely used for the purpose of detecting the presence of oversize pores or defects that can compromise the retention capability of a filter. The test can be destructive, as in a direct bacterial challenge performed during filter validation or as a manufacturing lot release test, but most often it is a nondestructive test to evaluate whether a filter unit is integral or contains a flaw. Because achieving a sterile effluent is of critical importance, sterilizing-grade filters are typically integrity-tested after assembly and prior to use, as well as again after use, as per industry and regulatory guidelines [1,2,3]. Several types of integrity tests for assessing the integrity of membrane filters for liquid filtration have been previously described, including particle challenge tests, the gas–liquid diffusion test, the bubble point test, and diffusion tests measuring tracer components [4,5,6,7,8,9,10,11,12]. Of these, variants of the gas–liquid diffusion test (gas–liquid pair combinations such as air–water, nitrogen–IPA, and others, along with the diffusive–forward flow and pressure hold–decay measurement methods) and bubble-point-type tests are most commonly used for sterilizing-grade filters [1,2,3,4,5,6,7,8,9,10,11,12,13]

Both the gas–liquid (often air–water) diffusion and the bubble point tests were conceptualized long ago—at least back to about 1973 for the gas–liquid diffusion test and 1894 for the bubble point test [14,15]. These tests have been refined over the years, and forms of these tests are extensively used today and have widespread acceptance in the bioprocessing industry. Both tests have also been endorsed by regulators, with FDA guidance stating, for example, that for sterilizing filters, “forward flow and bubble point tests… are two integrity tests that can be used” to detect any filter leaks [2]. Similarly, EU guidance states that the “integrity of the sterilised filter should be verified before use and should be confirmed immediately after use by an appropriate method such as a bubble point, diffusive flow or pressure hold test” [3].

As the purpose of these tests is to assess filter integrity, a key performance metric for these tests is their minimum detectable leak rate owing to filter defects or oversized pores. Indeed, for integrity tests used for filters, containers, films, etc., a commonly used metric for rating the sensitivity or defect detection capability of a test is the smallest-sized single defect that the test can reliably detect. For example, studies comparing the capability of various integrity tests for container closure systems or single-use bioprocess systems have based comparisons on the smallest-sized defect that the integrity test methods can reliably detect [16,17]. For the air–water diffusion test, the minimum detectable single defect size in a sterilizing-grade 10-inch (25.4 cm) cartridge filter has previously been estimated to be in the range of 5–20 µm, depending on the filter area, accuracy of the diffusive flow measurement, and variability in filter properties [11,18,19].

For the bubble point test, there are no known published studies on the minimum detectable defect size or the level of retention assurance provided by this test as applied to 10-inch sterilizing-grade cartridge filters. The bubble point test is often paired with the air–water diffusion test (these two tests complement each other) so that the combined defect detection sensitivity when both tests are run is at least that of the air–water diffusion test. However, in many cases, including in some recent studies pertaining to filter integrity testing, a bubble point test is the only test used to assess the integrity of a filter [20,21]. It is especially in these cases where knowledge of the defect detection sensitivity of the bubble point test is crucially important.

The bubble point test was initially developed as a manual visual test used for disk filters. In that format, defects or oversized pores can be detected in a wetted membrane covered with liquid, with the bubble point defined as “the lowest pressure at which a steady stream of bubbles rises…” [22]. Since the bubble point relates to the largest pore or set of pores in a membrane, many investigators have correlated membrane bubble point to its particle retention performance [23,24,25]. However, these types of studies have typically been conducted on small disk (often 47 mm) samples. For 10-inch cartridges or assemblies of 10-inch cartridges, a bubble point test is usually carried out using automated integrity testers (AITs). AITs are available from several vendors. However, there is no universal standard for the determination of bubble point in cartridge filters, and the methodologies and algorithms that are used are specific to AIT vendors. Therefore, the defect detection capability of a bubble point test (throughout this paper, the term “bubble point test” refers to the collection of methods as applied by different AITs) is also AIT-specific. Even within an AIT, user-adjustable bubble point recipe settings can impact the defect detection sensitivity of the test. Therefore, just as there is no universal standard for the determination of bubble point in cartridge filters, there is no universal answer to the question of what the defect detection sensitivity of the bubble point test is.

Many authors have considered the limitations of bubble point tests as applied to cartridge filters and assemblies of cartridge filters, but concerns have primarily focused on measurement accuracies and the subjectivity aspects that are involved in determining the bubble point [6,7,26,27,28]. For example, it has been pointed out that the bubble point measured on larger-area devices, such as 10-inch pleated cartridges that may contain the equivalent of 500–1000 47 mm disks in terms of filtration area, can be measured to be significantly different than on 47 mm disks due to several factors. One of these factors is that the absolute background diffusive flow rate through a membrane increases in proportion to the filter area, obscuring the transition between primarily diffusive flow and the onset of convective flow through pores that have been evacuated of the wetting fluid. This paper identifies and quantifies additional limitations of using bubble point tests to assess the integrity of sterilizing-grade cartridge filters that should be considered by filter users who opt to use a bubble point integrity test without pairing it with a gas–liquid diffusion test.

In this study, AITs from three different vendors are used to test two types of 10-inch sterilizing-grade filters for the purpose of quantifying the defect detection sensitivity of a bubble-point-only test (i.e., not paired with a gas–liquid diffusion test). Controlled leaks are created by diverting a portion of the inlet feed stream to the permeate or by creating laser hole defects of controlled sizes within the membrane pleat pack. The corresponding level of bacterial retention associated with laser hole defect size and, therefore, the minimum level of retention assurance afforded by the bubble point test are determined.

## 2. Theoretical Background

### 2.1. Bubble Point

The principles of bubble point testing have been described elsewhere and so are only briefly summarized here [4,5]. Microporous membranes fill their pores with liquid in accordance with the laws of capillary rise. By applying gas pressure, liquid can be forced out of the filter pores. The minimum pressure required to evacuate a pore is as follows:

(1) $$P=\frac{4k\cos\theta }{d}\sigma$$

where *P* is the applied gas pressure, *k* is the pore shape factor, *θ* is the angle of wetting, *σ* is the surface tension of the liquid, and *d* is the diameter of the pore. The removal of liquid from the largest pores creates a passageway through which bulk gas flow takes place. The minimum pressure at which this bulk flow through the membrane is detected is referred to as the bubble point. Since the bubble point indicates the largest membrane pore size, which can be correlated to bacterial retention, bubble point measurement can provide an indirect measure of retention.

**Figure 1:**
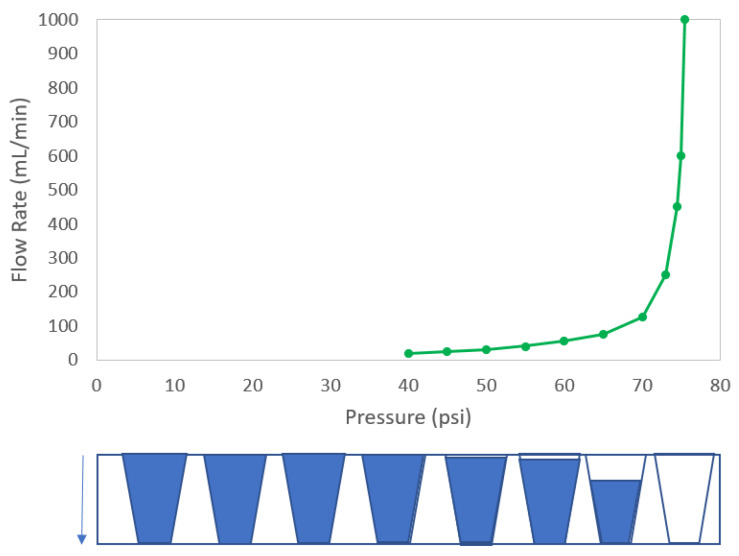
Hypothetical bubble point curve for an asymmetric membrane. Bottom illustration shows liquid level in idealized conical pores (representing the largest pores within the pore size distribution) corresponding to the pressure differential across the pores. Arrow indicates direction of applied pressure.

The determination of bubble point in cartridge filters involves applying gas pressure to a wetted membrane, starting at a pressure below the expected bubble point of the membrane and increasing the pressure continuously or in stepwise fashion. At pressures below the bubble point, a wetted membrane provides a liquid layer across which diffusive gas flow occurs in accordance with Fick’s law of diffusion, as expressed below:

(2) $$Q=\frac{A\varepsilon DS\left(P_{f}-P_{p}\right)}{\tau L}$$

where *Q* is the permeation flow rate; *A* is the membrane area; *ε* is the membrane porosity; *D* is the diffusivity of the gas in the liquid; *S* is the solubility coefficient of the gas; *P_f_* and *P_p_* are the feed and permeate side pressures, respectively; *τ* is the pore tortuosity; and *L* is the thickness of liquid in the membrane. As pressure is increased, diffusive flow increases linearly until either the liquid layer begins to thin or until the bubble point is reached, where robust bulk gas flow commences. For a funnel shaped pore (e.g., in an asymmetric membrane) pressure causes liquid to flow out of the funnel below the true bubble point (see Figure 1). Depending on the algorithm used to define the bubble point (discussed later in this paper), the bubble point derived from the hypothetical curve in Figure 1 would be within the range of about 70–75 psi.

At a pressure below the bubble point (typically at 80% or less of the expected minimum bubble point), a gas flow rate in excess of that predicted by Equation (2) or higher than a flow rate empirically established for an integral membrane is a signal for a defect. This is the basis of the gas–liquid diffusion test.

### 2.2. Effect of Defects

Methods for estimating the gas and liquid flow rates through a cylindrical defect in a membrane have been described elsewhere, and a similar approach was followed here [11,18,29]. If the gas flow in a pore defect is laminar and the gas velocity is below sonic velocity, gas flow rate can be estimated using the Hagen–Pouiselle equation for compressible fluids:

(3) $$Q=\frac{\pi d^{4}\left(P_{f}^{2}-P_{p}^{2}\right)}{256P_{p}\mu L}$$

where *µ* is the viscosity. For conditions where the gas flow is choked (gas velocity is limited by the sonic velocity), the flow rate can be estimated using Equation (4):

(4) $$Q=\frac{C_{d}\pi d^{4}P_{f}^{}v_{s}}{4P_{p}}$$

where *C_d_* is the discharge coefficient and *v_s_* is the sonic velocity of the gas. A measured excess gas flow rate (above the integral limit) can, therefore, be translated into a theoretical single pore defect size or multiple smaller pores.

The liquid flow rate through a cylindrical defect where the flow is laminar is described by the Hagen–Pouiselle equation for noncompressible fluids:

(5) $$Q=\frac{\pi d^{4}(P_{f}^{}-P_{p}^{})}{128\mu L}$$

For a cylindrical defect with a small (<~5) L/d ratio, the flow through the defect can be approximated as flow through an orifice:

(6) $$Q=\frac{C_{d}\pi d^{2}}{4\sqrt{1-\beta^{4}}}\sqrt{2\left(P_{f}-P_{p}\right)\rho }$$

where *β* is the ratio of the orifice diameter to the pipe diameter, and *ρ* is the fluid density. For a liquid flowing through a cylindrical membrane defect with a small L/d ratio, Equation (6) can be simplified as follows:

(7) $$Q=C_{v}d^{2}\sqrt{\left(P_{f}-P_{p}\right)}$$

where *C_v_* is an empirical orifice coefficient. For water at room temperature flowing through micromachined orifices, a *C_v_* value of about 0.028 m^3^s^−1^Pa^−0.5^ was found to provide a good fit to the data (data not shown).

The retentiveness of a membrane is often expressed as either a sieving value, *C_p_*/*C_f_*, or a log reduction value:

(8) $$LRV=Log_{10}\left[\frac{C_{f}}{C_{p}}\right]$$

where *C_f_* is the concentration of the retained species in the feed, and *C_p_* is the concentration of the retained species in the permeate (also known as the filtrate). The impact of a liquid leak on retention can be calculated using the following:

(9) $$LRV=Log\left(\frac{V_{T}}{V_{I}*10^{-LRV_{I}}+V_{d}}\right)$$

where *LRV_I_* is the *LRV* of the integral (defect-free) portion of the membrane, *V_I_* is the volume of feed passing through the integral portion of the membrane, *V_d_* is the volume passing through the defect, and *V_T_* is the total volume of feed passing through the filter. It was assumed that the concentration of the retained species in the liquid flowing through a defect was the same as that in the bulk feed, that is, the defect did not retain any of the species retained in the integral portion of the membrane. Since in this work all the defect holes created in the membranes were about 20 µm in diameter or larger and the bacteria used to assess filter retention were less than about 1 µm in any dimension, this was a reasonable assumption.

## 3. Materials and Methods

### 3.1. Membranes and Devices

The sterilizing-grade filtration devices used in this work are listed in Table 1. Two membrane types were evaluated: 0.2 µm asymmetric polyethersulfone (PES) and 0.2 µm symmetric polyvinylidene fluoride (PVDF) membranes. All the devices contained pleated membranes in commercially available formats consisting of a membrane, support materials, internal and external sleeves, and end caps. The devices had a nominal length of 10 inches (25.4 cm) and a diameter of approximately 2.7 inches (6.9 cm). These devices were further classified into two groups consisting of control devices and controlled defect devices. Control devices were commercially available devices free of defects. Controlled defect devices were devices containing a defect created in the membrane and installed into the device. A detailed explanation of the controlled defects in the membranes is described in the next section.

In addition to the 0.2 µm devices, pleated 10-inch (25.4 cm) cartridges containing symmetric PVDF 0.45 µm membranes and asymmetric PES 0.45 µm membranes were also evaluated. These 0.45 µm devices were tested for bubble point only to compare with the bubble points of the integral 0.2 µm devices.

### 3.2. Controlled Leaks and Defects

**Figure 2:**
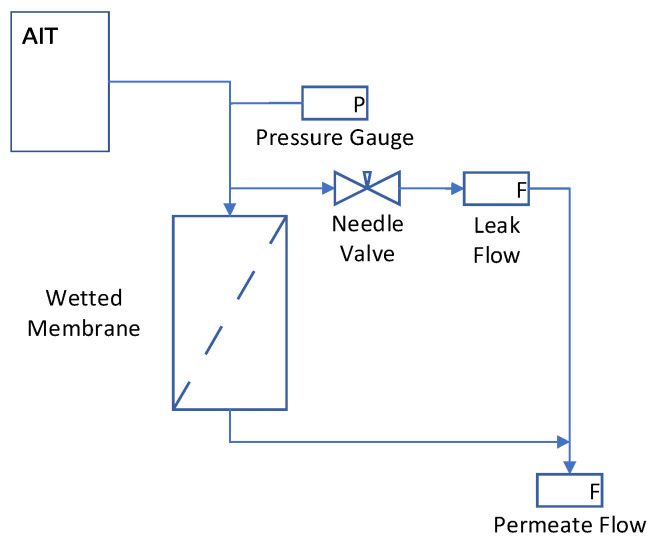
Schematic of controlled leak experimental setup.

Controlled leaks were used to quantify the capability of the bubble point test to detect leaks resulting from defects. Two methods were used to generate the controlled leaks: (1) installing a leak path from the upstream side of the filter to the downstream side of the filter and (2) creating defects of known sizes in the membrane. For method (1), a metering valve was used to control the leak flow rate from the feed side to the permeate side, as shown in Figure 2. The metering valve was adjusted to achieve a desired leak flow rate at 40 psi (276 kPa) and locked so that the leak rate increased in proportion to pressure as the pressure was stepped up during the bubble point test. Since this test could be run repeatedly on one device of known integral diffusive flow rate and bubble point, this method allowed for the precise determination of the impact of leak rate on bubble point. Mass flow meters (Cole-Parmer, Vernon Hills, IL model numbers 0–1 L/min MFM 32908-67 and 0–100 mL/min MFM 32908-59) were used to measure the diffusive flow and leak rates and a digital pressure gauge (GE, Leicester, UK, GE Druck DPI 104 100 PSIG S/N 2592309) were used to measure the inlet pressure to the filter.

**Figure 3:**
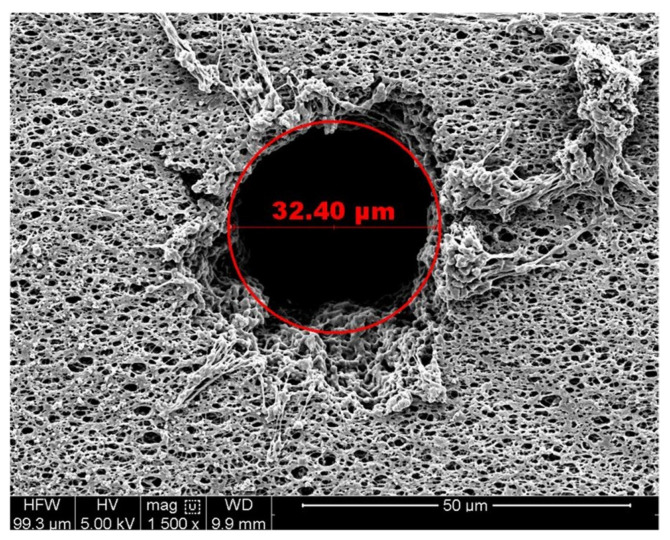
SEM image of a nominal 30 µm diameter laser hole in a SD-type membrane.

Defects in the membrane were generated by micromachining small holes by laser drilling. While “real-world” defects can encompass a variety of forms, laser-drilled holes are considered to be the most practical and controllable among various methods for creating artificial leaks in thin films and membranes and have often been used for this purpose [11,16,17,20,30,31]. Nominal defects between 20 µm and 100 µm in diameter were created using a UV laser (Resonetics, Nashua, NH). The holes were drilled after the membranes were pleated. The pleat pack was then assembled into a standard 10-inch (25.4 cm) cartridge format. Figure 3 shows an SEM image that is representative of a laser hole in a membrane.

### 3.3. Automated Integrity Testers

Commercially available AITs from three vendors were used in this study and were designated as VA, VB, and VC. All the testers were the most recent AIT versions offered by the vendors at the time of the study, and all of them were validated by the vendor for the determination of diffusive flow rate and bubble point of sterilizing-grade filters. They all worked on the principle of deriving the flow rate across a filter by measuring the rate of pressure decay on the upstream side of the filter, but the algorithms used for determining the bubble point from flow rate vs. pressure data were specific and, to some extent, proprietary to each AIT.

As noted, the bubble point test does not refer to a specific test protocol, which can be specific to the equipment used and operating choices made by the user. The purpose of this study was not to evaluate the capabilities of any particular piece of equipment with respect to bubble point testing but rather to quantify the range of defect detection sensitivities that filter users can typically expect when using commercially available AITs for determining the bubble point of sterilizing-grade filters. Therefore, the AITs used in this study are referred to only in generic terms.

Each AIT had its own set of user-adjustable operating parameters that could impact the bubble point test results. These parameters could include, for example, stabilization time, measurement time, and pressure step increment size. Default or vendor-recommended test parameters were used for all the bubble point tests. In some cases, more than one set of default settings were offered by the AITs, allowing, for example, faster test time in exchange for a minor compromise in test accuracy. The Results and Discussion section of this paper refers to different sets of default settings within an AIT as default A1 and default A2 (A1 provides for faster test time compared to A2) for vendor A, default B for vendor B, and default C for vendor C.

### 3.4. Test Procedure

#### 3.4.1. Bubble Point and Diffusion Tests

In preparation for diffusion and bubble point testing, the filters were first wetted with RO water at 7.5 L/min for 5 min at 40 psi (276 kPa) back pressure or with a solution of 70:30 IPA:water. Free fluid was then drained from the devices and connected to an AIT. For the water-wetted filters, the feed gas source was compressed air, and for the IPA-and-water-wetted filters, the feed gas source was compressed nitrogen. The input values for the bubble point test recipe (such as pressure step increment, for example) were specific to each AIT, but in each case, either the AIT default values or vendor-recommended values were used for each test.

Identical test procedures were used for integral devices and for the devices containing controlled defects. For the devices tested in accordance with Figure 2, the filters were first tested without any leak (needle valve closed) to establish a baseline integral bubble point value. The filters were then rewetted and tested again with a preset leak through a needle valve. This procedure was repeated for several leak rates.

Although ascertaining the sensitivity of the gas–liquid diffusion test was not within the scope of this study, a diffusion test was run at the manufacturer-specified test pressure for each filter with the respective AIT that was used to run the bubble point test for that filter. The diffusion test served to confirm the leak flow rate that was applied to the filters.

#### 3.4.2. Retention Tests

To assess the retention capability of the filters, bacterial retention was measured in accordance with ASTM F838-20. A membrane is defined as “fully retentive” and, therefore, sterilizing grade if it retains a challenge of >10^7^ colony-forming units (CFUs) per square centimeter of membrane area for an extremely small bacterium (Brevundimonas diminuta). For filters that experienced passage of bacteria into the filtrate, LRV was calculated as per Equation (8).

## 4. Results and Discussion

### 4.1. Controlled Bypass Leak

**Figure 4:**
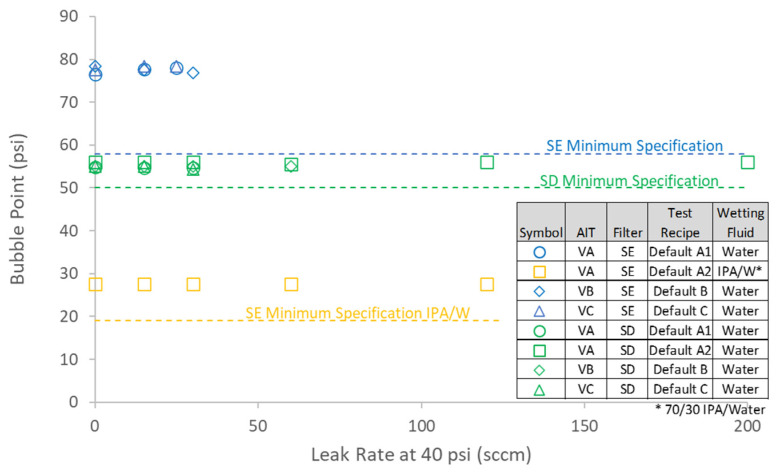
Effect of leak rate on reported bubble point. Default or vendor-recommended values were used for each AIT.

The reported bubble point by the AIT as a function of leak rate at 40 psi (276 kPa) per setup of Figure 2 is shown in Figure 4 for both the SE and SD filters. The reported bubble point was invariant with leak rate up until the point where an AIT reported an invalid or failed test. Above a certain flow rate (or rate of upstream pressure decay), the AIT algorithm considered the flow rate to be either a “gross leak” or too high to accurately measure (resulting in a failed test). While the term “gross leak” is replete in the integrity test literature and in AIT operating manuals, it is rarely, if ever, quantitatively defined. The criteria for what constitutes a gross leak—which are not made explicit in any user manuals of the AITs used in this study—appeared to be different among the different AITs.

**Figure 5:**
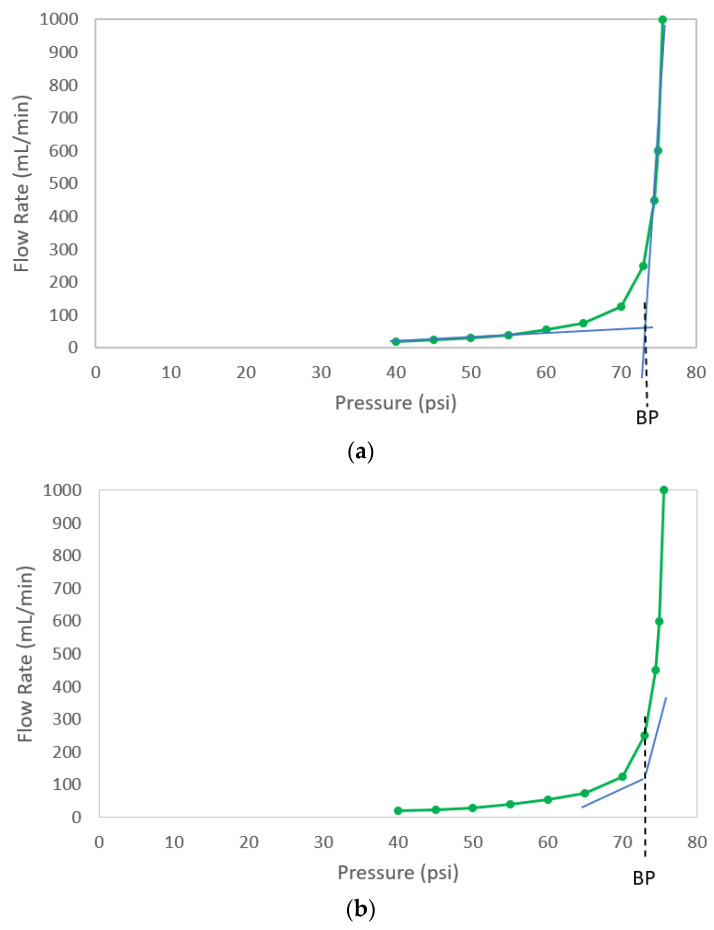

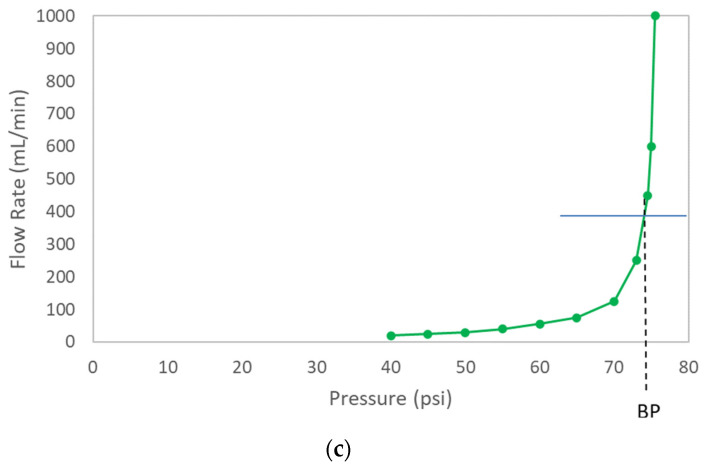
Determination of bubble point from flow rate vs. pressure spectra: (**a**) tangent method; (**b**) slope change threshold method; (**c**) threshold flow rate method.

To understand why the reported bubble point did not change with leak rate, the method used to determine bubble point must be elucidated. Consider the bubble point curve of Figure 1 that is reproduced in Figure 5. There are several methods that can be used to extract the bubble point from this curve. One is the so-called tangent method [32]. In this method, the linear portions of the curve representing diffusive flow through the liquid layer of a membrane and the linear portion of the curve representing convective flow through pores evacuated of water are fitted to straight lines, as shown in Figure 5a. The pressure at the intersection of these curve tangents is the bubble point. The bubble point for the curve in Figure 5a per this methodology would be about 74 psi (510 kPa).

Another method for determining bubble point from flow rate–pressure spectra is to identify the pressure at which the slope change of the bubble point curve exceeds an established threshold. For asymmetric membranes where thinning of the liquid layer can occur (resulting in a nonlinear increase in flow rate with increasing pressure), the slope change threshold that is indicative of the transition between diffusive flow and the onset of convective flow can be confounded. Depending on the slope change threshold value, the bubble point of the filter depicted in Figure 1 would be in the 70–75 psi (483–517 kPa) range, as illustrated in Figure 5b.

A simpler method for defining the bubble point is the pressure at which the flow rate exceeds an established value. For example, if the threshold flow rate value is set to 500 mL/min, then the bubble point in Figure 5c would be 75 psi (517 kPa). Other methods are possible, but they generally attempt to determine the pressure at which the flow rate transitions from primarily diffusive flow through the liquid-filled pores to primarily convective flow through pores evacuated of liquid. Differences in results among bubble point tests tend to arise not from differences in the interpretation of the flow rate vs. pressure curves, but rather from differences in how these bubble point curves are generated [6,7,26,27,28].

Almost regardless of which of the three bubble point determination methods described above was used, the leak rate had a minimal impact on the reported bubble point (the flow rate vs. pressure curves that were the basis of the bubble point determinations in Figure 4 are shown in Appendix A). The curves associated with the leaks were nearly indistinguishable from the integral curves in terms of the bubble point for the tangent method, the slope change method, and the threshold diffusive flow method. This behavior was irrespective of filter type and has been observed with filters from various filter vendors (data not shown). Owing to the published correlations for membrane disks that show decreasing bacterial LRV with decreasing bubble point, there is a perception by many that the bubble point of a cartridge filter decreases with increasing leak rate. Contrary to this notion, the data showed that the reported bubble point practically did not change with increasing leak rate up until the point that the flow rate (measured by rate of upstream pressure decay) reached a level at which a valid bubble point could not be determined (a failed test) or the leak was characterized as a “gross” or unacceptable leak per the AIT recipe or algorithm.

**Figure 6:**
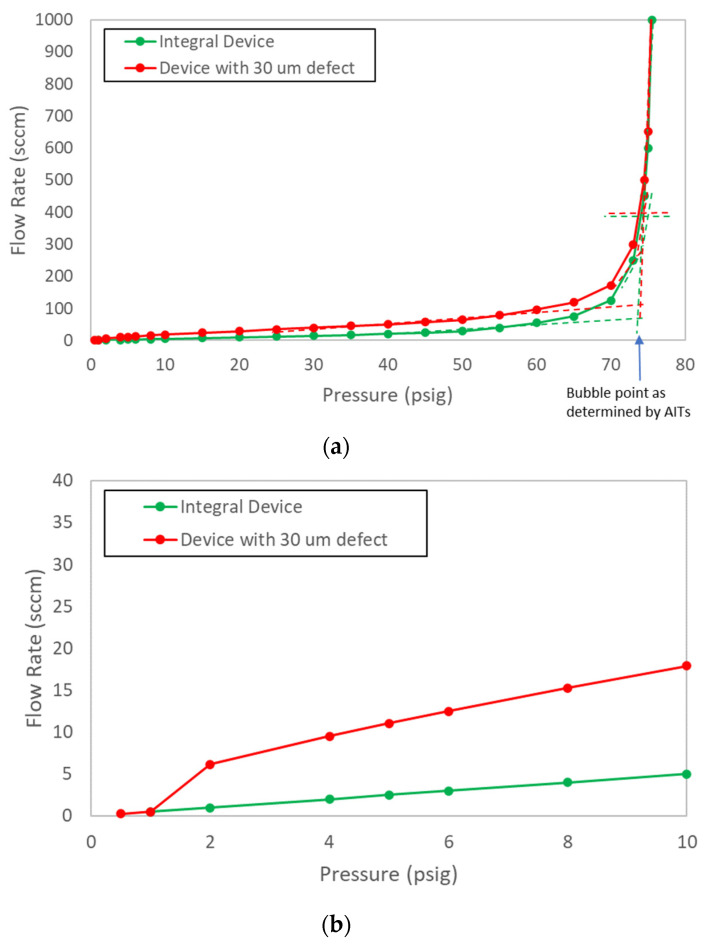
Hypothetical flow rate vs. pressure curve of Figure 5 with hypothetical 30 µm diameter defect: (**a**) full curve extended to 1 psi (7 kPa); (**b**) portion of curve below 10 psi (70 kPa).

Probing further into the lack of sensitivity of reported bubble point to leak rate, the equivalent defect size associated with a leak rate and the expected bubble point for that defect size could be estimated using Equations (4) and (1). An air flow rate of 30 sccm at 40 psi (276 kPa) was consistent with short, cylindrical hole of about 30 µm in diameter. In accordance with Equation (1), the air–water bubble point of this hole should be about 1.4 psi (9.7 kPa). This means that, to accurately determine the “true” bubble point of the filter, the flow rate vs. pressure curve would need to be initiated at a pressure below 1.4 psi (9.7 kPa), at which point an inflection in the flow rate vs. pressure curve could be detected, at least in principle. Figure 6a shows the hypothetical curve of Figure 5 extended down to 1 psi (7 kPa), along with a superimposed curve representing a device containing a hypothetical 30 µm diameter defect. As previously discussed, based on the algorithms used by the AITs to determine bubble point, there were no significant differences in bubble point between the integral devices and the devices containing the 30 µm defect for any of the three bubble point determination methods of Figure 5. Figure 6b focuses on the 0–10 psig (0–69 kPa) range of Figure 6a. This plot reveals that, if the flow rate vs. pressure drop curve was initiated at 1 psi (7 kPa) or less, it would be possible to ascertain the “true” bubble point in this filter as less than 2 psi (14 kPa).

However, for sterilizing-grade filters where the bubble point is typically about 50 psi (345 kPa) or higher, it is impractical to routinely start at such a low pressure. Some authors proposed assessing the slope of flow rate vs. pressure curves at pressures below the bubble point specification of the filter, suggesting that the presence of a defect renders the curve non-linear [18]. However, at a typical starting pressure of 40 psi (276 kPa), the flow rate through an orifice-like defect is choked, such that the flow rate increases linearly with pressure, just as the diffusive flow rate through the integral portion of a membrane increases linearly with increasing pressure (absent thinning of the liquid layer with increasing pressure). Therefore, the slope of a flow vs. pressure curve prior to the onset of primarily bulk flow through pores evacuated of liquid is not affected by such a defect.

### 4.2. Filters with Controlled Laser Hole Defects

**Figure 7:**
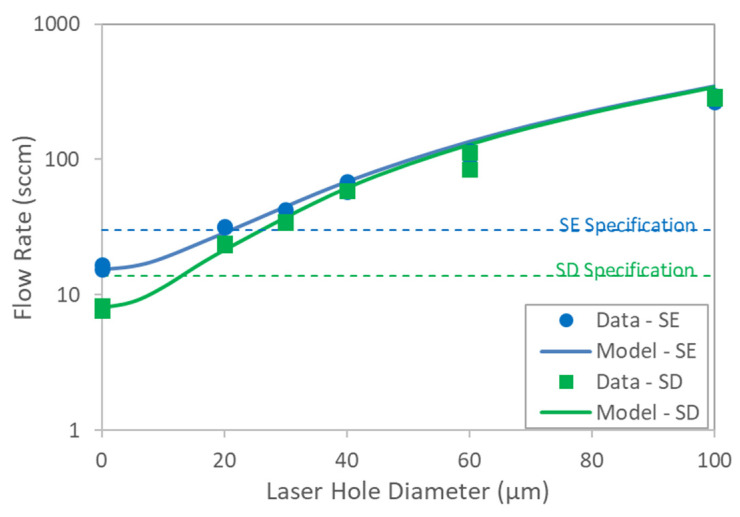
Flow rate of 40 psi (276 KPa) through SE- and SD-type filters as a function of laser hole diameter. Diffusion specifications indicated in plot are from filter vendors.

**Figure 8:**
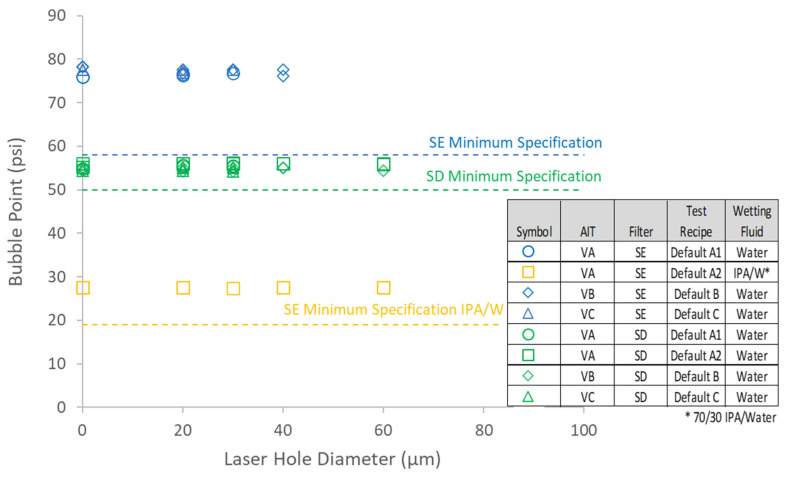
Effect of controlled laser hole defect on reported bubble point. Filters were tested in duplicate.

To directly assess the impact of defects in sterilizing-grade filters on the bubble point as reported by AITs, both SD- and SE-type filters containing controlled laser hole defects were tested with each of the three AITs. The measured air diffusion as a function of defect size is summarized in Figure 7 and shows good agreement with the model predictions. Figure 8 shows bubble point as a function of defect size for each filter type and AIT (using the bubble point recipe default settings for each AIT), as well as for two different wetting fluids. Consistent with the controlled bypass leak results (Section 4.1), bubble point was nearly invariant with defect size up until the point where the tester either ascertained a gross leak or was unable to determine a valid bubble point. Although each AIT had its own proprietary bubble point determination algorithm, there were no significant differences among the bubble point values reported by the different AITs for either filter type. The largest defect size that resulted in a passing reported bubble point did depend on the combination of filter type and AIT vendor, although every device that contained a laser hole defect of 30 µm or smaller showed passing bubble point values for all the AITs. In some cases, devices containing defects as large as 60 µm exhibited passing bubble point values. For defect sizes larger than about 60 µm, the AITs reported either an invalid result or a gross leak, but in no case did a defect result in a depressed bubble point value.

**Figure 9:**
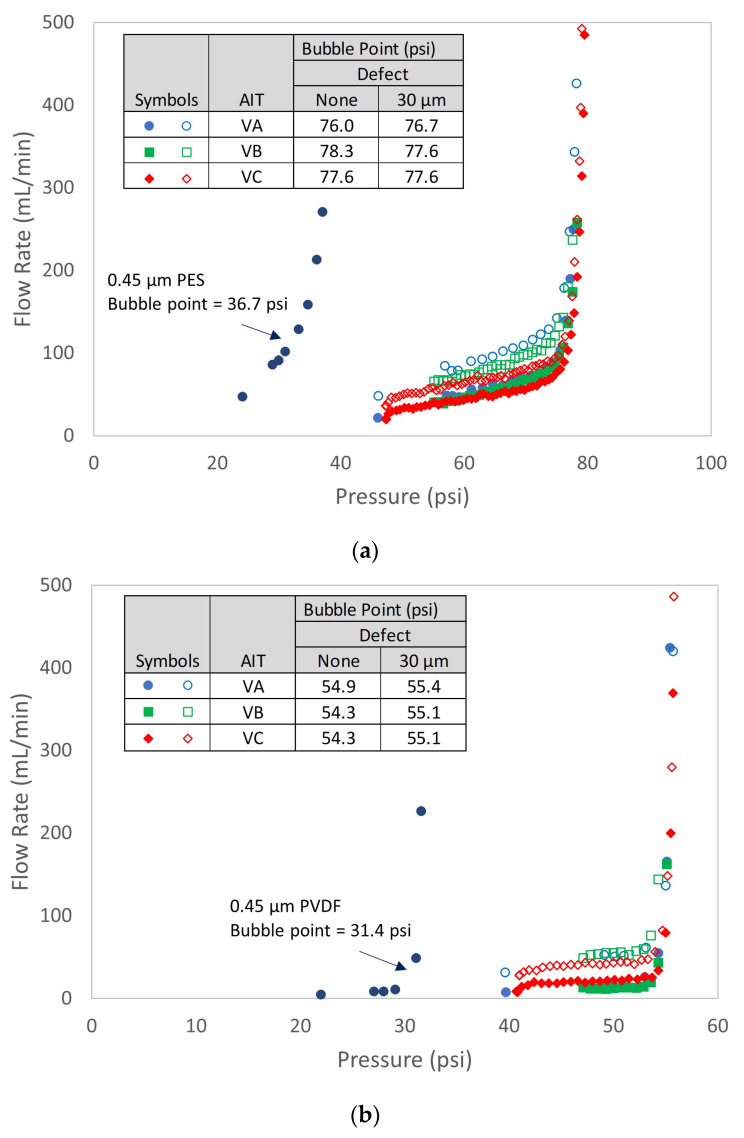
Flow rate vs. pressure comparison between a 0.2 µm defect-free device and a device containing a 30 µm laser hole, along with defect-free 0.45 µm device: (**a**) SE filter; (**b**) SD filter.

For both SE and SD devices, Figure 9 shows a comparison of the flow rate vs. pressure curves between defect-free devices and devices that contained a 30 µm laser hole. Similar to the effect of controlled leaks shown in Appendix A, the leak through 30 µm laser holes shifted the flow rate vs. pressure drop curves upward but did not impact the reported bubble point of any of the AITs. It was noted that the bubble point values of the integral nominal 0.45 µm membrane cartridges (measured using the VA AIT) were significantly lower, as expected, compared to the 0.2 µm membrane cartridges. This confirms that, although the bubble point values as reported by AITs were insensitive to defects, they were sensitive to rated membrane pore size.

While the laser hole defects did not result in depressed bubble points (where values were reported), the air diffusion rates of the filters containing laser holes were elevated compared to the integral filters. The diffusion value at the pressure where a filter had a diffusion specification could, in principle, be used as an indicator of filter integrity. However, the bubble point test algorithm does not necessarily measure the diffusion rate at the pressure at which the diffusion specification is stated by the filter vendor (assuming the filter has a diffusion specification) since the starting pressure and pressure increments vary from vendor to vendor and recipe to recipe. Furthermore, for AITs to complete a bubble point test in a reasonable amount of time, the measurement of diffusive flow rate at each pressure step is usually an abbreviated version of a full diffusion test for which the specified test pressure is precisely controlled and the criteria for equilibration of the diffusive flow rate at the test pressure may be more stringent. Therefore, the intermediate diffusion flow rates in a bubble point test at pressures that are lower than the bubble point cannot be relied on for determining filter integrity; a formal diffusion test at the vendor-specified conditions needs to be conducted in conjunction with the bubble point test.

If the measured flow rate in a bubble point test is very high, the filter is deemed as failing due to a gross leak or an invalid test result. However, as noted, there are no established, quantified criteria for what constitutes a gross leak or invalid test result. Filter vendors do not provide specifications for gross leaks, so the user of an AIT must rely on the algorithm used by the AIT, the details of which are not typically transparent to the user. This means that, while an AIT-based bubble point test ensures that an installed filter is of the proper pore size, it provides an uncertain level of retention assurance for the filter.

### 4.3. Impact of Defects on Bacterial Retention

**Figure 10:**
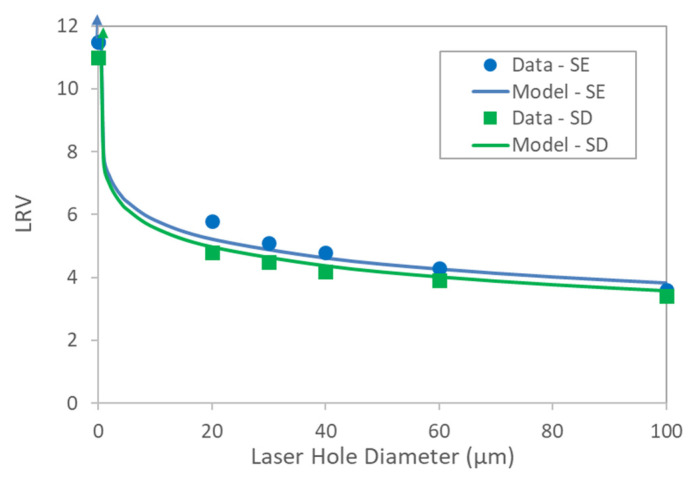
Effect of laser hole defect size on bacterial retention. Arrows indicate that no bacteria were detected in the filtrate.

**Figure 11:**
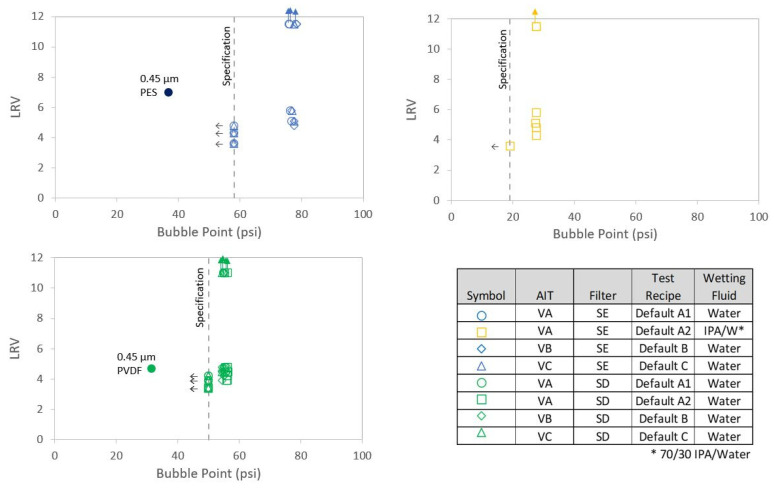
Relationship between reported bubble point and LRV. Upward-pointing arrows indicate no bacteria detected in the filtrate. Leftward-pointing arrows indicate that the AIT either reported a gross leak or invalid result, not a quantified bubble point below the filter specification.

Both the SD and SE devices that contained laser hole defects, along with the control integral devices, were tested for bacterial passage in accordance with ASTM F838-20. LRV as a function of defect size is shown in Figure 10. For both sets of data, the model predictions were in good agreement with the measured values. The model lines assumed an integral membrane bacterial LRV > 11–11.5, which was about the assay upper limit of the retention test. Since sterilizing-grade filter cartridges that contained laser hole defects as large as 60 µm in diameter exhibited passing AIT bubble point values, filters with LRV values as low as about 4–4.5 passed the bubble point test, as can be seen in Figure 10. All the filters that contained 30 µm holes passed the bubble point test for all the AITs, and those filters exhibited LRV values of about 4.5–5. Since reported bubble point was invariant with defect size until an invalid or gross leak result was reported, there was no correlation between LRV and bubble point, as shown in Figure 11 for filters with the same pore size ratings. Cartridge filters containing membranes with different pore size ratings (e.g., 0.1 µm, 0.2 µm, 0.45 µm, 0.65 µm, etc.) did show a relationship between bacterial retention and bubble point, and as can be seen from Figure 9 and Figure 11, the integral 0.45 µm cartridge filters did exhibit lower bubble point and lower LRV values compared to the corresponding integral 0.2 µm cartridge filters. Interestingly, the 0.45 µm PES filters had much lower reported bubble points but equal or higher LRV values than the corresponding 0.2 µm filters that contained laser hole defects. While pore size impacted the reported bubble point of the AITs for cartridge filters, the presence of defects in the filters (where valid bubble points were reported) did not. All the filters that contained laser hole defects exhibited air diffusion values that were above the vendor specifications and, therefore, failed that test.

As previously noted, “real-world” defects in membrane filters are not necessarily cylindrical in shape as with the controlled laser hole defects in this study. The relationship between defect size, leak rate, and bacterial retention depends on the specific geometry of a defect or defects. The results presented here, however, demonstrate that defects in cartridge membrane filters that were large enough to easily allow the passage of bacteria could escape detection by bubble point tests.

## 5. Conclusions

In conjunction with a multitiered approach for assuring the bacterial retention of sterilizing-grade filters, bubble point tests are widely used for assessing filter integrity. Originally developed long ago as a manual visual test for filter disks, bubble point tests have been adapted to cartridge filters with the use of automated integrity testers. While bacterial retention performance has been correlated with bubble point on disk filters, there is a lack of published data * (* As remarked in a book published in 1987 but still pertinent as of this writing, “one hears stories that defects in cartridges, induced by penetration by a pin, may or may not be detectable by either diffusional air flow or bubble point measurements, depending on what area of the pleating has been penetrated. Surely, however, if such experiments had been performed, their results would have been deemed worthy of formal reporting” [33].) showing a correlation between retention performance and bubble point for cartridge filters containing controlled defects. In this study, it was found that bubble point as determined by three different AITs was practically invariant with defect size up to the point where an AIT either determined that the filter contained a “gross leak” or was not able to produce a valid bubble point result. The fact that reported bubble point did not decrease with increasing defect leak rate is contrary to the perception of many but consistent with an understanding of the methodologies used in AITs to ascertain bubble point. A “gross leak” causes a filter to fail the bubble point test, but there is no quantified definition of “gross leak”, which is dependent on the algorithm used by each AIT to make that determination. The algorithms used by AITs are usually not transparent to the user, and in any case, while filter vendors typically provide air–water diffusion specifications, they do not provide “gross leak” specifications for their filters.

While the level of retention assurance of sterilizing-grade filters provided by the bubble point test as determined by AITs is filter- and AIT-specific (and within an AIT, potentially user-setting-specific), the results presented here showed that leaks from laser hole defects as large as 60 µm in diameter in 10-inch (25.4 cm) sterilizing-grade pleated cartridge filters and results for bacterial LRV as low as about 4 could escape failing a bubble point test. All the filters that contained laser hole defects that were 30 µm in diameter or smaller and that exhibited an LRV as low as about 4.5, passed the bubble point test for all three AITs used in this study. This is the first known published study that quantified the bacterial retention assurance of sterilizing-grade 10-inch filter cartridges afforded by AIT-administered bubble point tests. Although the bubble point test had relatively low sensitivity to filter leaks, it did serve to confirm the pore ratings of the installed filters. The gas–liquid diffusion test, which is not sensitive to pore size at pressures below the bubble point, is better able to detect filter defects. Pairing these two complementary tests is a generally recommended practice to give a more comprehensive evaluation of filter integrity. For users of sterilizing-grade cartridge filters that rely solely on the bubble point test to assess filter integrity in critical applications, the information presented here may spur reconsideration of that approach when feasible.

## Figures and Tables

**Table 1:** Sterilizing-grade pleated cartridge filters.

Filter Designation	Membrane	Filtration Area (m^2^)	Air/Water Bubble Point Specification psi (kPa)
SE	0.2 µm PES	0.54	≥58 (400)
SD	0.2 µm PVDF	0.69	≥50 (345)

**Figure A1:**
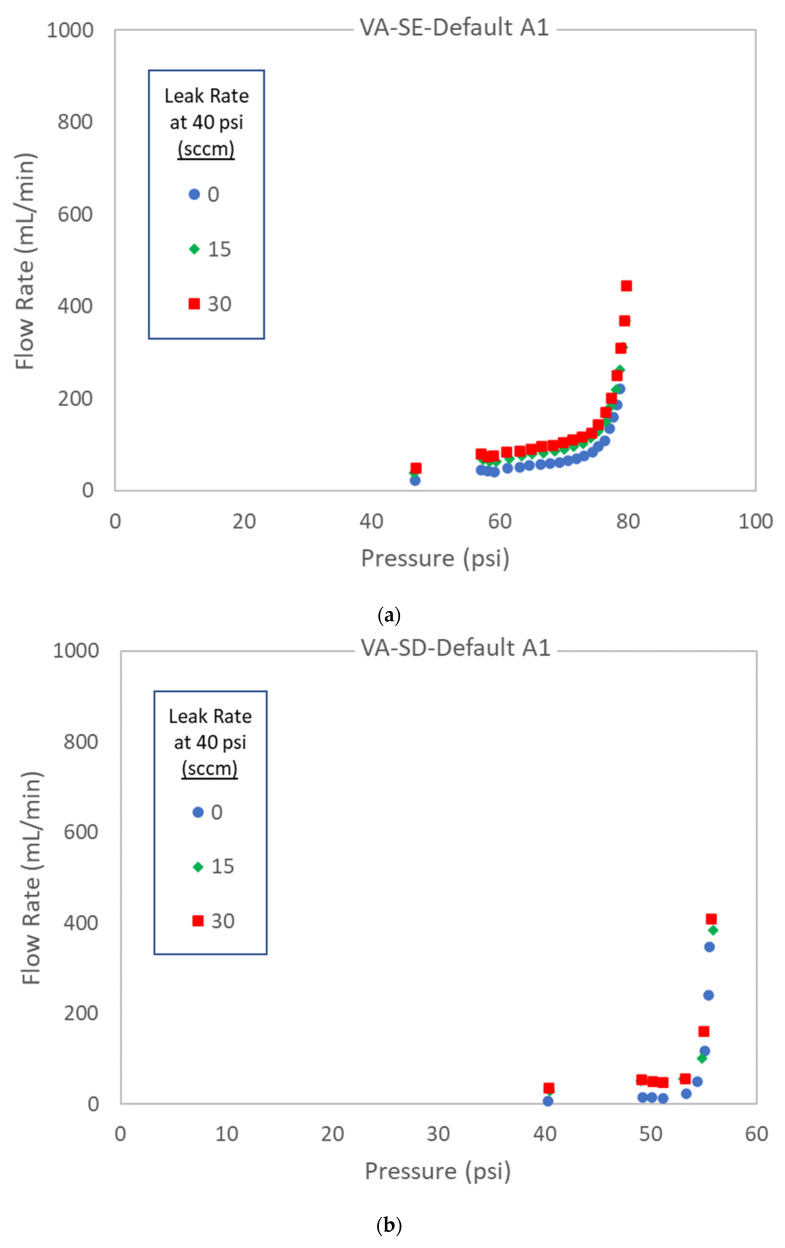

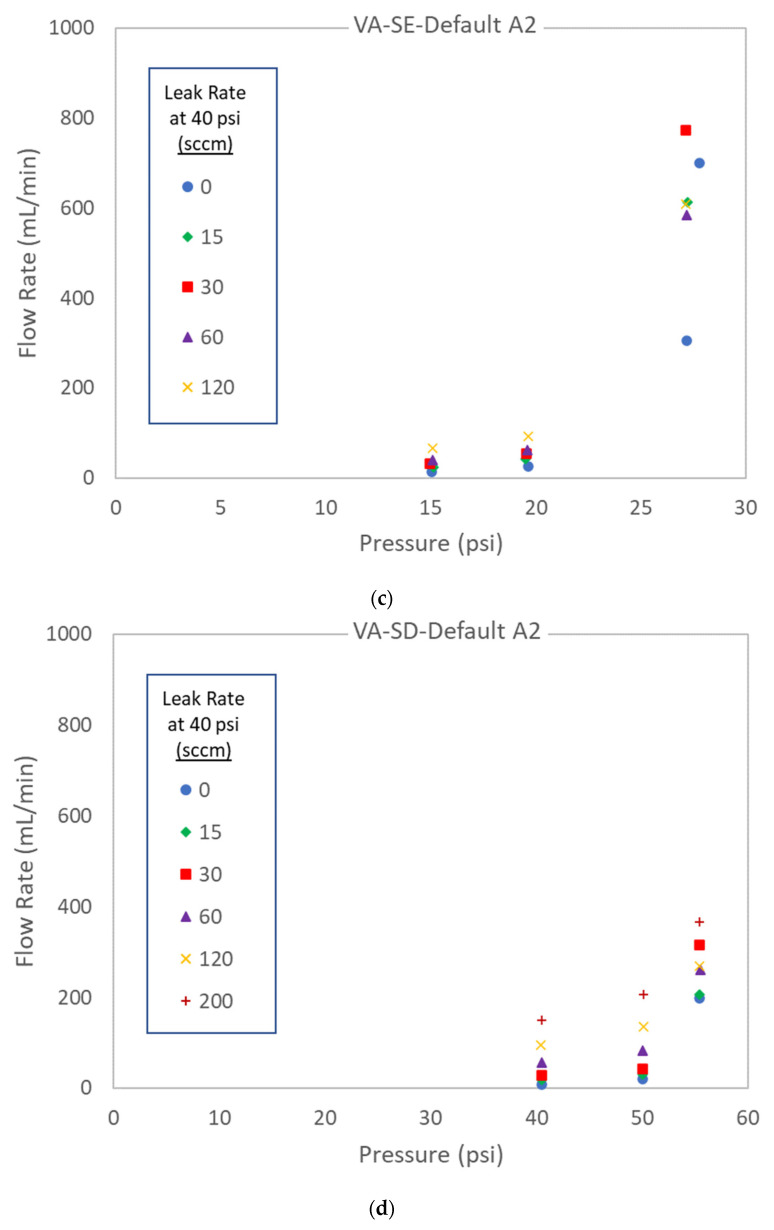

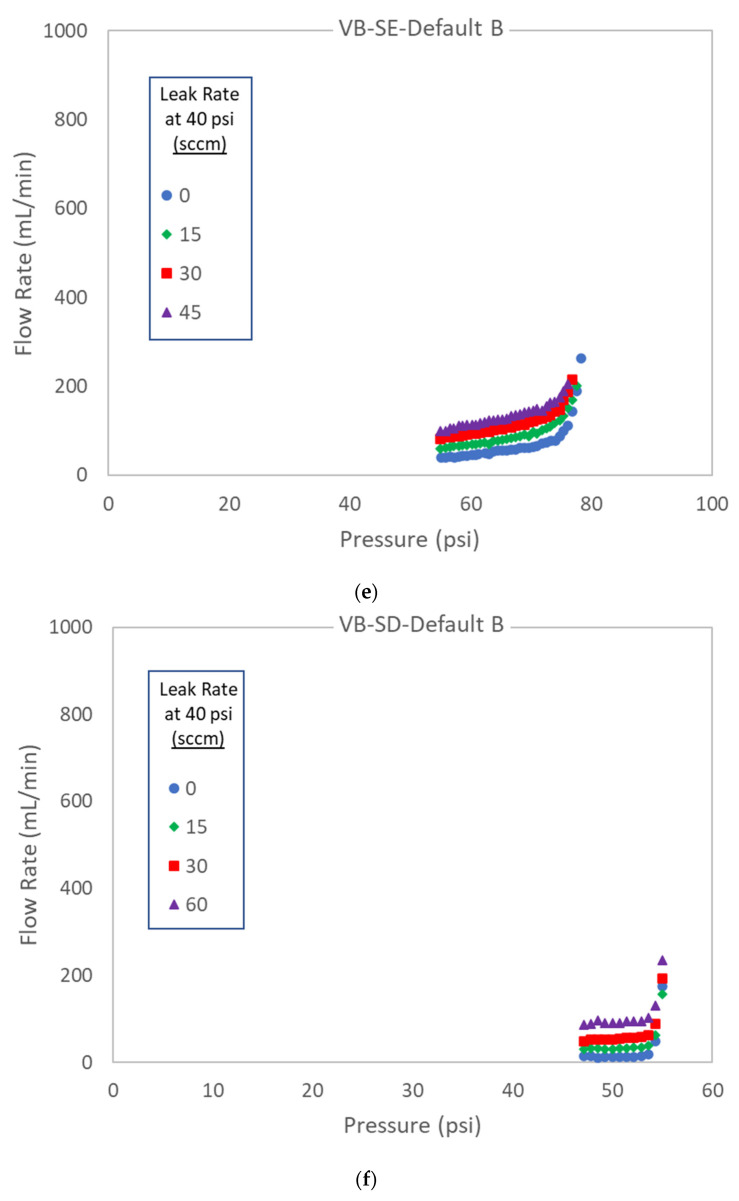

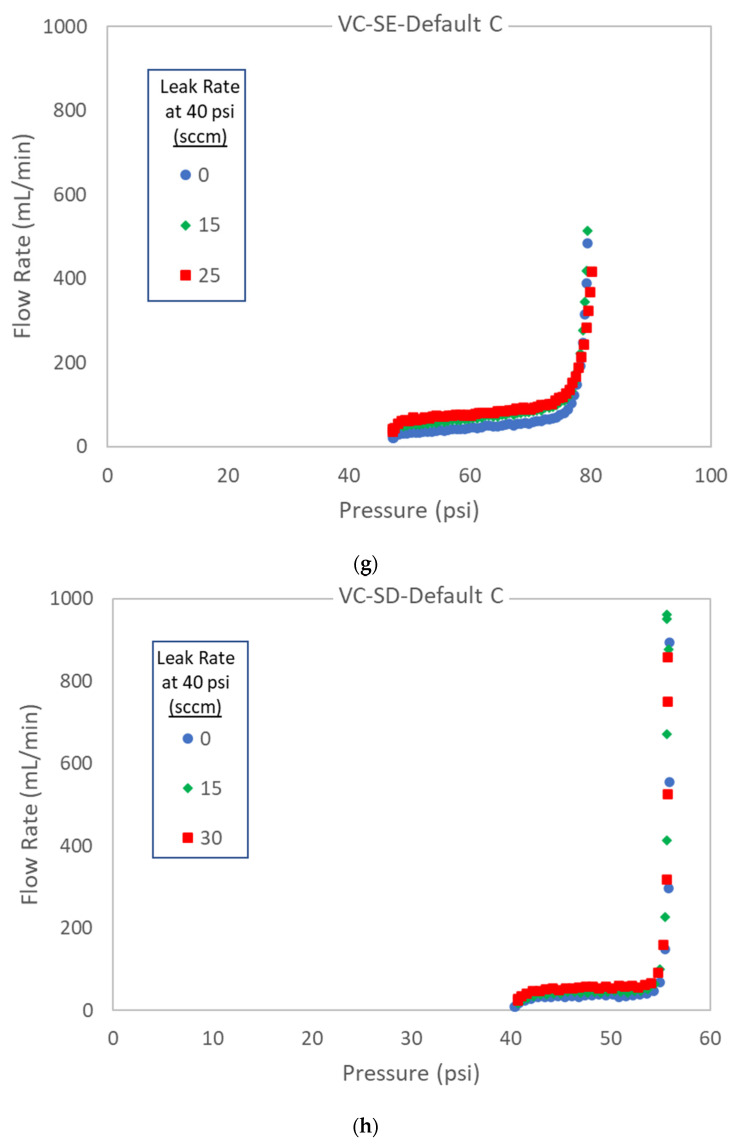
Effect of leak rate on bubble point curves: (**a**) vendor A, SE membrane, default A1 recipe; (**b**) vendor A, SD membrane, default A1 recipe; (**c**) vendor A, SE membrane, default A2 recipe; (**d**) vendor A, SD membrane, default A2 recipe; (**e**) vendor B, SE membrane, default B recipe; (**f**) vendor B, SD membrane, default B recipe; (**g**) vendor C, SE membrane, default C recipe; (**h**) vendor C, SD membrane, default C recipe.

## Data Availability

The data are not publicly available due to confidentiality.

## Appendix A

The flow rate vs. pressure curves that were the basis of the bubble point determinations in Figure 4 are shown in Figure A1. The leak rate had little impact on the pressure at which there was an inflection in the bubble point curves and, therefore, little impact on reported bubble points. For all the curves in Figure A1, the pressure at which the slope made an abrupt upward transition was practically unaffected by the leak.
